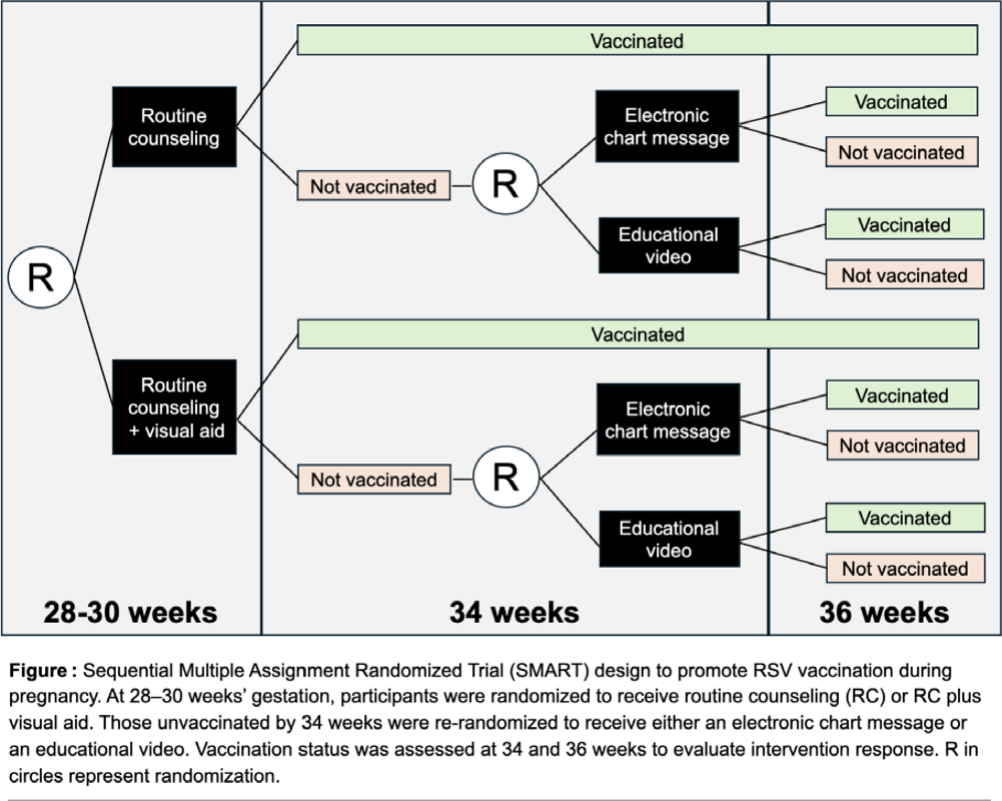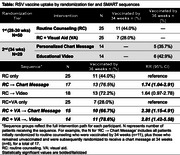# Oral Concurrent Session 4 – Equity, Public Health, and Public Policy: Thursday, February 12, 2026 1:00 PM – 3:15 PM

**DOI:** 10.1002/pmf2.70161

**Published:** 2026-02-09

**1:00 PM – 1:15 PM**

## 45 | Impact of a Substance Use Screening Program on Racial Disparities in Urine Toxicology Testing

Mariam Naqvi^1^; Amin Tavakoli^2^; Camelita Thrift^3^; Allison Henry^4^; Jennifer Astasio^1^; Naomi H. Greene^3^; Sarah Kilpatrick^1^

^1^Department of Obstetrics and Gynecology, Cedars‐Sinai Medical Center, Los Angeles, CA; ^2^Department of Obstetrics and Gynecology, Cedars‐Sinai Medical Center, Los Angeles, CA, CA; ^3^Department of Obstetrics & Gynecology, Cedars‐Sinai Medical Center, Los Angeles, CA; ^4^Department of Pediatrics, Cedars‐Sinai Medical Center, Los Angeles, CA

**Objective**: To evaluate whether implementing a standardized substance use screening program reduced racial disparities in urine toxicology (Utox) testing among delivery admissions.

**Study Design**: We conducted a retrospective cohort study of delivery admissions at a single academic center from June 2019 to June 2025. To address known disparities in Utox testing, a standardized iPad‐based screening tool using a modified 4Ps screen was implemented Feb–May 2023, with full rollout after June 2023. Screening responses triggered protocolized Utox testing based on a predefined algorithm (Figure). Clinical and demographic predictors of Utox testing were compared pre‐ and post‐implementation using chi‐square and t‐tests. Logistic regression with predictor × era interaction terms assessed changes in associations with testing**;** final models adjusted for age, parity, and insurance.

**Results**: Among 36,656 admissions, 216 (0.59%) underwent Utox testing: 181 (0.80%) pre‐implementation and 32 (0.26%) post‐implementation (*p* < 0.001). Pre‐implementation, Black patients accounted for 26.8% of tests but only 7.0% of the population (*p* < 0.001). Post‐implementation, Black patients represented 6.3% of tests and 6.5% of the population (*p*  =  0.91). The testing rate for Black patients declined from 3.15% to 0.51% (*p* < 0.001). The proportion of tested patients with public insurance declined from 65.7% to 53.1%, though not significantly (*p*  =  0.19). In unadjusted models testing for effect modification by implementation period, race (*p* = 0.02 for Black vs White; interaction OR 0.17, 95% CI 0.05–0.66) and advanced maternal age (*p* = 0.001; interaction OR 1.14, 95% CI 1.06–1.23) showed significant interactions. In adjusted models, the disparity in testing by race significantly decreased post‐implementation (interaction aOR for Black vs White 0.17, 95% CI 0.05–0.66; *p* = 0.01). Among those tested, positive Utox screens did not differ significantly by period (43.2% vs. 56.3%, *p*  =  0.11).

**Conclusion**: Implementation of a standardized substance use screening tool was associated with a significant reduction in racial disparities in Utox testing at delivery.

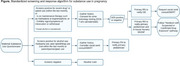

**1:15 PM – 1:30 PM**

## 46 | **Experiences of** Discrimination **and Adverse Pregnancy Outcomes—A Secondary Analysis of the EMBRACE Comparative‐Effectiveness Trial**

Daisy León‐Martínez^1^; Deborah Karasek^2^; Brittany D. Chambers Butcher^3^; Cinthia Blat^4^; Venise Curry^5^; Patience A. Afulani^6^; Charles E. McCulloch^7^; Miriam Kuppermann^8^

^1^Division of Maternal‐Fetal Medicine at the University of California San Francisco, University of California San Francisco, CA; ^2^Oregon Health & Science University‐Portland State University School of Public Health, Portland, OR; ^3^Department of Human Ecology, College of Agricultural and Environmental Sciences, University of California, Davis, Davis, CA; ^4^Department of Obstetrics, Gynecology and Reproductive Sciences, University of California, San Francisco, San Francisco, CA; ^5^Central Valley Health Policy Institute at California State University, Fresno, Fresno, CA; ^6^Department of Obstetrics, Gynecology and Reproductive Sciences, University of California, San Francisco, Universiy of California San Francisco, CA; ^7^University of California, San Francisco, University of California San Francisco, CA; ^8^Department of Obstetrics, Gynecology and Reproductive Sciences, University of California, San Francisco, University of California San Francisco, CA

**Objective**: To evaluate the association of race‐ and ethnicity‐based discrimination and adverse pregnancy outcomes (APO).

**Study Design**: Secondary analysis of EMBRACE, a randomized trial comparing depression, care experiences, and preterm birth (PTB, <37 weeks) among Medicaid‐eligible pregnant people assigned to enhanced group (eGPC) or enhanced individual prenatal care (eIPC).

Primary outcome: Composite measure of APO including gestational diabetes (GDM), hypertensive disorders of pregnancy (HDP), PTB, placental abruption, small for gestational age, and stillbirth. Secondary outcomes were GDM, HDP, and PTB individually. Outcome data were collected via hospital records and participant interviews. Discrimination was assessed using two validated self‐report measures: the 10‐item Experiences of Discrimination (EOD) scale at study entry, and the 9‐item Discrimination in Medical Settings (DMS) scale at 30–34 weeks’ gestation. Scores were standardized for comparability.

Associations between a 1‐standard deviation increase in discrimination scores and APO (any vs none) were estimated using generalized estimating equations (GEE) logistic regression models with robust standard errors, adjusting for race, ethnicity and comorbidities (pre‐gestational diabetes, chronic hypertension, pre‐pregnancy BMI >30 kg/m^2^). Covariates were selected by backwards stepwise regression. Subgroup analyses were conducted for participants self‐identifying as Latine or Black.

**Results**: Among 652 participants (*n* = 281 eGPC, *n* = 371 eIPC), self‐identified race and ethnicity was 7.4% Black, 72.2% Latine, 11.2% White. Overall, 34.3% experienced an APO (18.3% HDP, 10.4% GDM, 10.7% PTB). Increasing experiences of healthcare discrimination were associated with higher odds of APO, HDP, and PTB. Significant associations persisted for APO and PTB after adjustment. Remaining analyses were not significant after adjustment (Figure 1). Analyses in the Black subgroup were limited by small sample size.

**Conclusion**: Racism in healthcare setting may contribute to poor perinatal outcomes. Ongoing efforts to address this are critical.

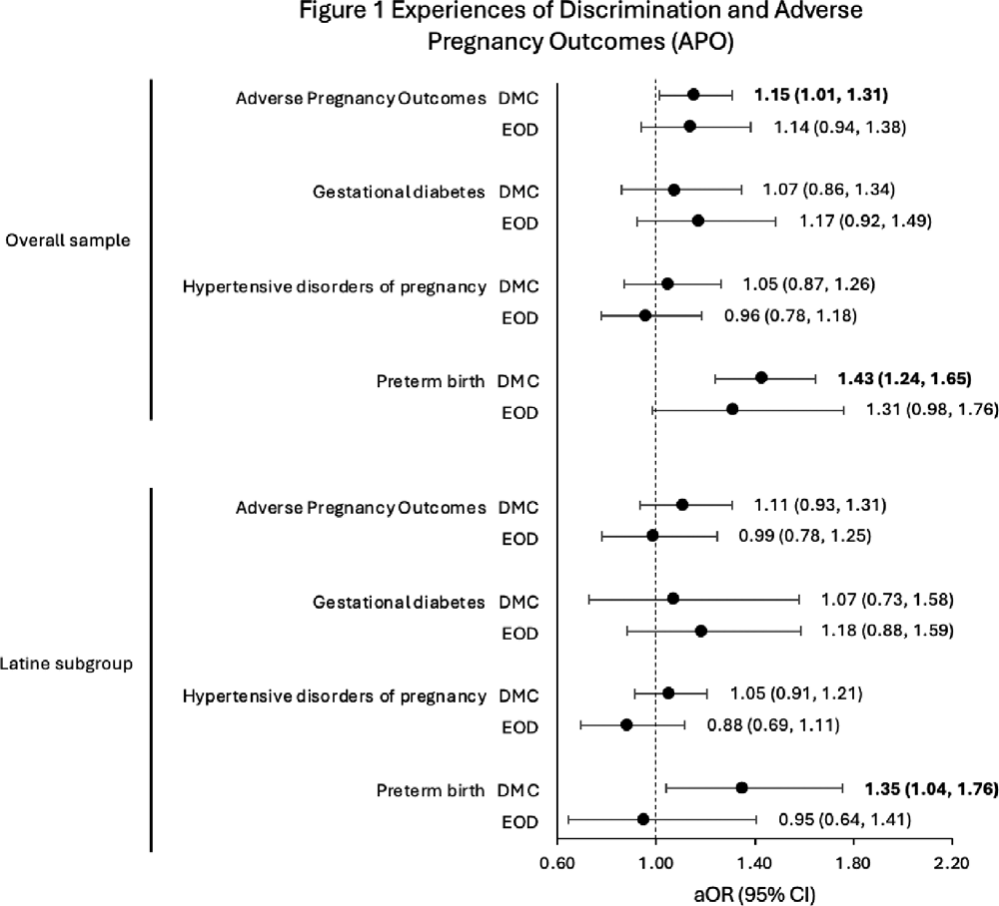

**1:30 PM – 1:45 PM**

## 47 | **Exploring Neighborhood‐Level Indicators of Social Disadvantage Associated With Prenatal Inflammation**

Sarah L. Kaplan^1^; Samantha P. Gottlieb^2^; Frances Kincaid^3^; Estefania Espinosa^4^; Kennedi Williams^4^; Jiafeng Li^5^; Madeline R. Meirhaeghe^6^; Abigail L. Aron^7^; Mary Akel^5^; Leena B. Mithal^8^; Tonia Branche^5^; Stephanie A. Fisher^9^

^1^Ann & Robert H. Lurie Children's Hospital of Chicago, Atlanta, GA; ^2^Ann & Robert H. Lurie Children's Hospital of Chicago, Lurie Children's Hospital, Chicago, IL, IL; ^3^Northwestern University, Northwestern University, IL; ^4^Northwestern University, Chicago, IL; ^5^Ann & Robert H. Lurie Children's Hospital of Chicago, Chicago, IL; ^6^Ann & Robert H. Lurie Children's Hospital of Chicago, LIncolnshire, IL; ^7^Ann & Robert H. Lurie Children's Hospital of Chicago, Ann & Robert H. Lurie Children's Hospital of Chicago, IL; ^8^Ann & Robert H. Lurie Children's Hospital; Northwestern University Feinberg School of Medicine, Chicago, IL; ^9^Northwestern University Feinberg School of Medicine, Chicago, IL

**Objective**: To evaluate the association of neighborhood‐level indicators of social disadvantage with inflammation in pregnancy.

**Study Design**: We performed a secondary analysis of data from the prospective Chicago Perinatal Origins of Disease (CPOD) cohort study of pregnant people with singleton gestations at an urban academic center. Co‐primary exposures were overall Social Vulnerability Index (SVI) ranking (CDC, 2022) and Neighborhood Deprivation Index (NDI) quintile (American Community Survey, 2013–2017), assessed at the U.S. census tract level based on participant addresses, with their subdomains secondarily assessed (Tables 1–2). Outcomes were log‐transformed concentrations of plasma inflammatory cytokines (IL1β, IL2, IL6, IL8, IL10, IFNγ, TNFα) and cytokine‐induced cell adhesion molecules (ICAM1, VCAM1), measured via Meso Scale Discovery immunoassay. Student's t‐tests compared mean differences in biomarker concentrations, and linear regression models generated beta (ß)‐coefficients, adjusted for pre‐pregnancy body mass index (BMI).

**Results**: Among 118 participants, 99 had plasma samples collected at a median 28.7 weeks’ gestation (IQR 26.1, 32.4). Fifty participants (50%) identified as non‐White. Median pre‐pregnancy BMI was 25.7 kg/m^2^ (IQR 22.3, 28.2). The overall SVI ranking was not associated with the assessed biomarkers. Higher NDI quintile was associated with lower VCAM1 concentrations (mean difference −0.19 pg/mL; *p*‐value 0.03). SVI sub‐scores for household characteristics (adj. ß −0.26; 95% CI −0.47, −0.05) and housing type/transportation (adj. ß 0.30; 95% CI 0.03, 0.56) were associated with VCAM1, while the SVI sub‐score for racial and ethnic minority status was positively associated with IL1β (adj. ß 1.20; 95 % CI 0.11, 2.29). Individual NDI sub‐scores were not associated with the biomarkers assessed.

**Conclusion**: VCAM1 and IL1β concentrations in maternal plasma varied by social vulnerability and neighborhood deprivation in the CPOD cohort, suggesting neighborhood‐level social stressors influence endothelial activation and inflammatory signaling in pregnancy.

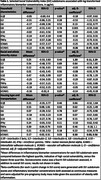

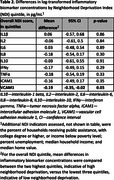

**1:45 PM – 2:00 PM**

## 48 | **State Abortion Restrictions and Maternal Deaths in the United States: 2005–2023**

Marie C. Anderson^1^; Hooman A. Azad^2^; Dana Goin^2^; Uma M. Reddy^3^; Mary E. D'Alton^4^; Erika E. Levi^5^; Lisa Nathan^3^

^1^Columbia University, New York, NY; ^2^Columbia University, Columbia University, NY; ^3^Columbia University Irving Medical Center, New York, NY; ^4^Division of Maternal‐Fetal Medicine, Department of Obstetrics and Gynecology, Columbia University Irving Medical Center, New York, NY; ^5^Columbia University, Columbia Univeristy, NY

**Objective**: The federal right to abortion in the US was protected by *Roe v. Wade* (1973) until the precedent was overturned by *Dobbs v. Jackson Women's Health* (2022). However, state‐level restrictions eroded access to abortion, especially in the 2000s‐2010s. This study aims to investigate the effect of state‐level abortion restrictions on maternal deaths in the US.

**Study Design**: Case‐level data on deaths in US women ages 15–54 years (2005–2023) were collected from the CDC's National Vital Statistics System. Ten common state‐level abortion laws were catalogued using LexisNexis. Random‐effects negative binomial models compared the effect of individual restrictions on maternal deaths, controlling for state‐level factors. A quasi‐experimental difference‐in‐difference model determined the increased number of deaths attributable to having stringent abortion restrictions (≥5 restrictions, top tercile of states); this form of analysis largely isolates the impact of an intervention.

**Results**: 22,380 pregnant/postpartum women died in the US from 2005–2023. Violence, unintentional drug overdose, and cardiovascular disease were the 3 leading causes. The number of abortion restrictions increased from average 2.7 to 5.3 restrictions per state from 2005–2023. In 2005, only 5 states were considered “most restrictive” (≥5 restrictions); in 2023, 27 states had this designation. (Figure 1)

Six of ten abortion restrictions were associated with higher rates of maternal death. Four restrictions were associated with higher rates of violent death. A sensitivity analysis during the pre‐*Dobbs* era had similar findings. States with ≥5 abortion restrictions had higher rates of maternal deaths from any cause, violent deaths, and cardiovascular disease. (Table 1)

**Conclusion**: Abortion is a safe alternative to continuing pregnancy. Restriction of abortion at the state level is strongly associated with increased rates of maternal death in the United States. Further restriction of abortion after *Dobbs* may result in higher rates of maternal death in coming years.

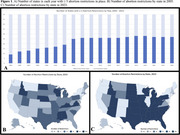

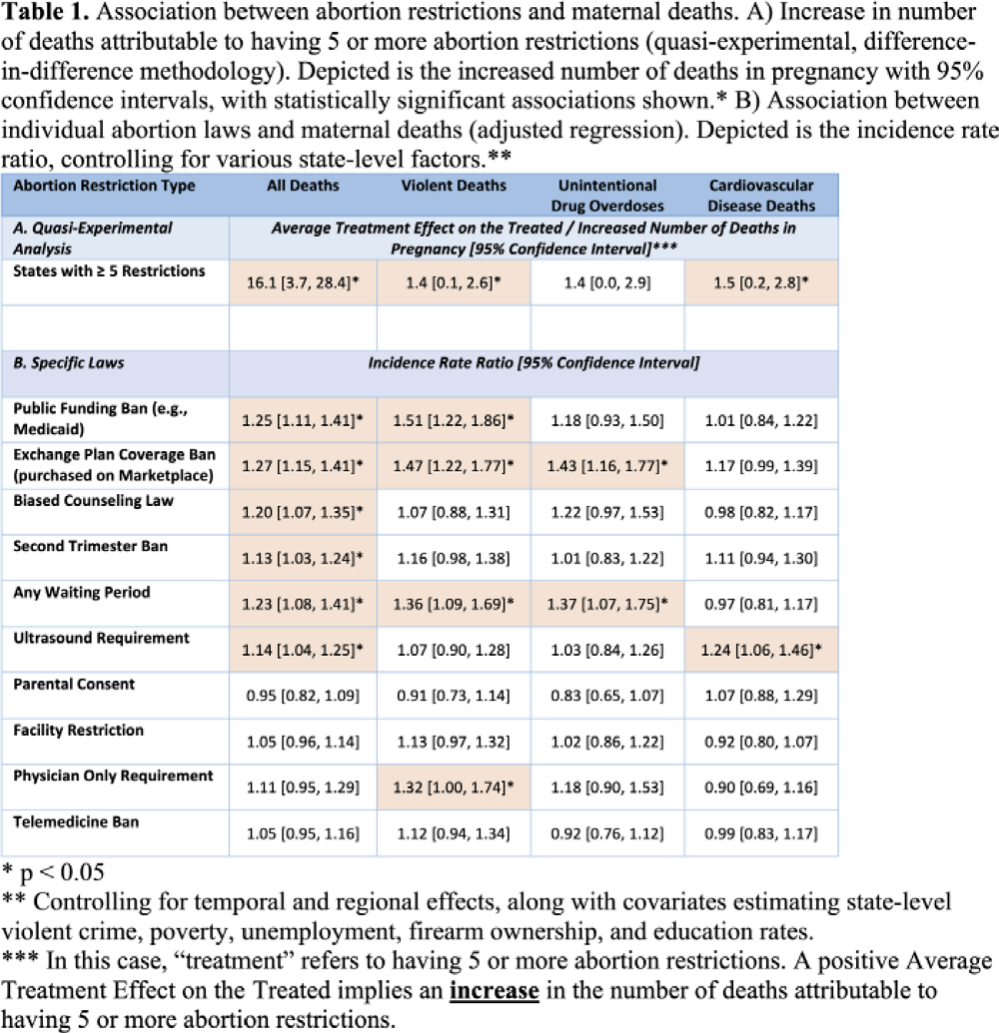

**2:00 PM – 2:15 PM**

## 49 | **Association Between Trial of Labor and Vaginal Birth After Cesarean Odds and Hospital‐Level Racial Segregation**

Max Jordan Nguemeni Tiako^1^; Chong Zhang^2^; Jaewhan Kim^2^; Adebayo Adesomo^3^; Michelle P. Debbink^4^

^1^University of California, Los Angeles (UCLA), Los Angeles, CA; ^2^University of Utah, University of Utah, UT; ^3^Utah Health, Utah Health, UT; ^4^University of Utah, Salt Lake City, UT

**Objective**: Racial segregation of obstetric services is often associated with worse quality indicators. However, individuals with a prior cesarean delivery (CD) have greater odds of a subsequent vaginal birth (VBAC) at hospitals serving a high (vs low) proportion of Black people. We hypothesized the difference may be attributable to differing rates of trial of labor after cesarean (TOLAC) by hospital type. We sought to assess the relationship between the proportion of births to Black individuals and attempting TOLAC or successful VBAC among those attempting.

**Study Design: R**etrospective cohort study of term deliveries with a prior cesarean from the US Nationwide Inpatient Sample, 2017‐2019. We excluded abnormal presentation, stillbirth, multiple gestation, or delivery at hospitals with <10 births. Primary outcomes were TOLAC and VBAC, defined using validated ICD codes. Primary exposure was Black serving hospital (BSH) status; hospitals were categorized by the proportion of births to Black people, divided into high (highest 5% of hospitals), medium (6–25%), or low (all others). We used inverse propensity score‐weighted, multivariable hierarchical logistic regression to assess relationships. Survey weights were used to create nationally representative estimates.

**Results**: 1,734,919 deliveries were included. 19.7% (*n* = 341,165) had a TOLAC and 227,775 (13.1%) a VBAC. Compared to low BSHs, patients at medium (aOR 1.13; 95% CI 1.07–1.19) and high (aOR 1.51; 95% CI 1.36–1.67) BSHs had higher TOLAC odds. Among TOLAC attempts, medium (aOR 1.08, 95% CI 1.01–1.16) and high (aOR 1.24; 95% CI 1.07–1.43) BSHs also had higher VBAC odds. At medium and high BSH, odds of TOLAC or VBAC did not differ by race, but Black patients (vs White) at low BSH had lower VBAC odds (aOR 0.88, 95% CI 0.78–0.98) (Figure).

**Conclusion**: High BSHs often perform worse on quality indicators, but we found higher odds of attempting TOLAC and no racial differences in VBAC rates when compared to low BSHs. Our findings add nuance to understanding obstetric services segregation and may inform efforts to reduce maternal morbidity disparities.

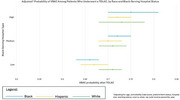

**2:15 PM – 2:30 PM**

## 50 | **Patient Navigation and Postpartum Patient‐Reported Outcomes Among Low‐Income Birthing People: A Randomized Controlled Trial**

Sydney L. Raucher^1^; Brittney R. Williams^1^; Joseph M. Feinglass^1^; Ying Cheung^1^; Laura Diaz^1^; Ka'Derricka Davis^1^; Michelle A. Kominiarek^1^; William A. Grobman^2^; Lynn M. Yee^1^

^1^Northwestern University Feinberg School of Medicine, Chicago, IL; ^2^Warren Alpert Medical School of Brown University, Providence, RI

**Objective**: To evaluate whether a patient navigation (PN) program improves patient‐reported outcomes (PROs) regarding perceived health status in the first year after birth.

**Study Design**: This pre‐planned analysis of a randomized trial evaluated whether postpartum (PP) PN improved self‐reported health among low‐income birthing people. Eligible people (≥16 years, English‐ or Spanish‐speaking, public insurance) were randomized 1:1 (>30 weeks’ gestation) to receive usual care or PP PN. PN participants were assigned to a trained lay navigator who offered non‐clinical assistance (e.g., social needs assessment, appointment coordination, emotional support) through one year PP. Researchers administered PRO measures selected to reflect health status at enrollment, 4–12 weeks (w) PP, and 11–13 months (m) PP. PROs were scored using standard methods. Differences between groups at each interval were determined using bivariable tests. Effect modification by health literacy (HL) status was found to exist for patient activation (*p* = 0.02 at 4–12 w) but not for other PROs; therefore, stratified analyses by HL status were performed for patient activation.

**Results**: From 1/2020 to 6/2023, 405 individuals were randomized (203 PN, 202 usual care), of whom 89.4% were retained through one year (Figure 1). Most participants identified as non‐Hispanic Black (50%) or Hispanic (41%). At enrollment, participants reported an overall high degree of well‐being, and PROs were similar in each group. At 4–12 w and 11–13 m, there was no significant difference between groups in any PRO evaluated, including global health, self‐efficacy, informational support, stress, PP knowledge and preparedness, care satisfaction, and depressive symptoms (Table 1). At 4–12 w, those with inadequate HL who received PN demonstrated improved patient activation (β 3.8, 95% CI 1.2–6.4, *p* = 0.004); patient activation did not differ (β −1.0, 95% CI −3.9 to 1.8, *p* = 0.48) for those with adequate HL.

**Conclusion**: Although a PP PN program did not improve PROs among low‐income people in the first year after birth, PN may improve patient activation for those with inadequate HL.

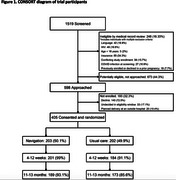

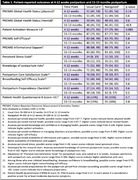

**2:30 PM – 2:45 PM**

## 51 | **Maternal Dietary Trajectories and Urinary Analyte Signatures Associate With Preterm Birth**

Michael D. Jochum, Jr.^1^; Cynthia Shope^2^; Melissa A. Suter^3^; Kjersti M. Aagaard^4^; Enrico R. Barrozo^3^

^1^Baylor College of Medicine, Houston, TX; ^2^Baylor College of Medicine, Baylor College of Medicine, TX; ^3^Baylor College of Medicine and Texas Children's Hospital, Houston, TX; ^4^HCA, Houston, TX

**Objective**: Preterm birth (PTB) is a leading cause of neonatal morbidity and long‐term disease risk, yet the role of environmental and dietary exposures in PTB occurrence remains unclear. Toxicants such as polycyclic aromatic hydrocarbons (PAHs) and microplastics accumulate in placentae and are elevated in PTB, but the impact of timing, sources, and dietary modifiers is unknown. We hypothesized that longitudinal urinary analyte measures could predict PTB and that maternal diet would mediate these relationships.

**Study Design**: We analyzed dietary data and longitudinal biospecimens from the BaBs cohort (NCT02392650), including 103 PTB and 367 term pregnancies. Mass spectrometry (HHEAR network) quantified 1230 analytes from maternal urine samples (*n* = 932; ≥2 per participant; median interval first sample‐to‐delivery: 20.6 weeks). Models integrated clinical and dietary metadata, including 2,129 survey measures. Diet index scores were generated using validated methods.

**Results**: Slopes (per‐subject rates of change across gestation) for multiple maternal urinary analytes and dietary scores were significantly associated with both dietary quality and PTB. Dietary patterns diverged by PTB status (Figure 1A), with Mediterranean Diet Score (MDS) declining steeply in PTB (interaction *p* = 0.016). At 28 weeks, PTB pregnancies had lower Alternate Healthy Eating Index (AHEI), fiber adequacy, and MDS (Figure 1B–D). Unsupervised clustering of analyte slopes revealed three groups, with Cluster 1 defined by a PAH, plasticizer, and metabolite signature. Cluster 1 was associated with a five‐week reduction in gestational age (Figure 2A–C; FDR < 0.05). Higher AHEI, MDS, and dietary diversity increased gestational age by 1.6–3.2 weeks, while increased AHEI and MDS interactions (+6 weeks) offset Cluster 1 risk; higher plastic exposure amplified risk (interaction −6.5 weeks).

**Conclusion**: Lower dietary quality and higher toxicant levels were independently and jointly associated with shorter gestational age. These results highlight the need to further evaluate maternal urinary analytes as PTB biomarkers and maternal diet as a modifiable risk factor.

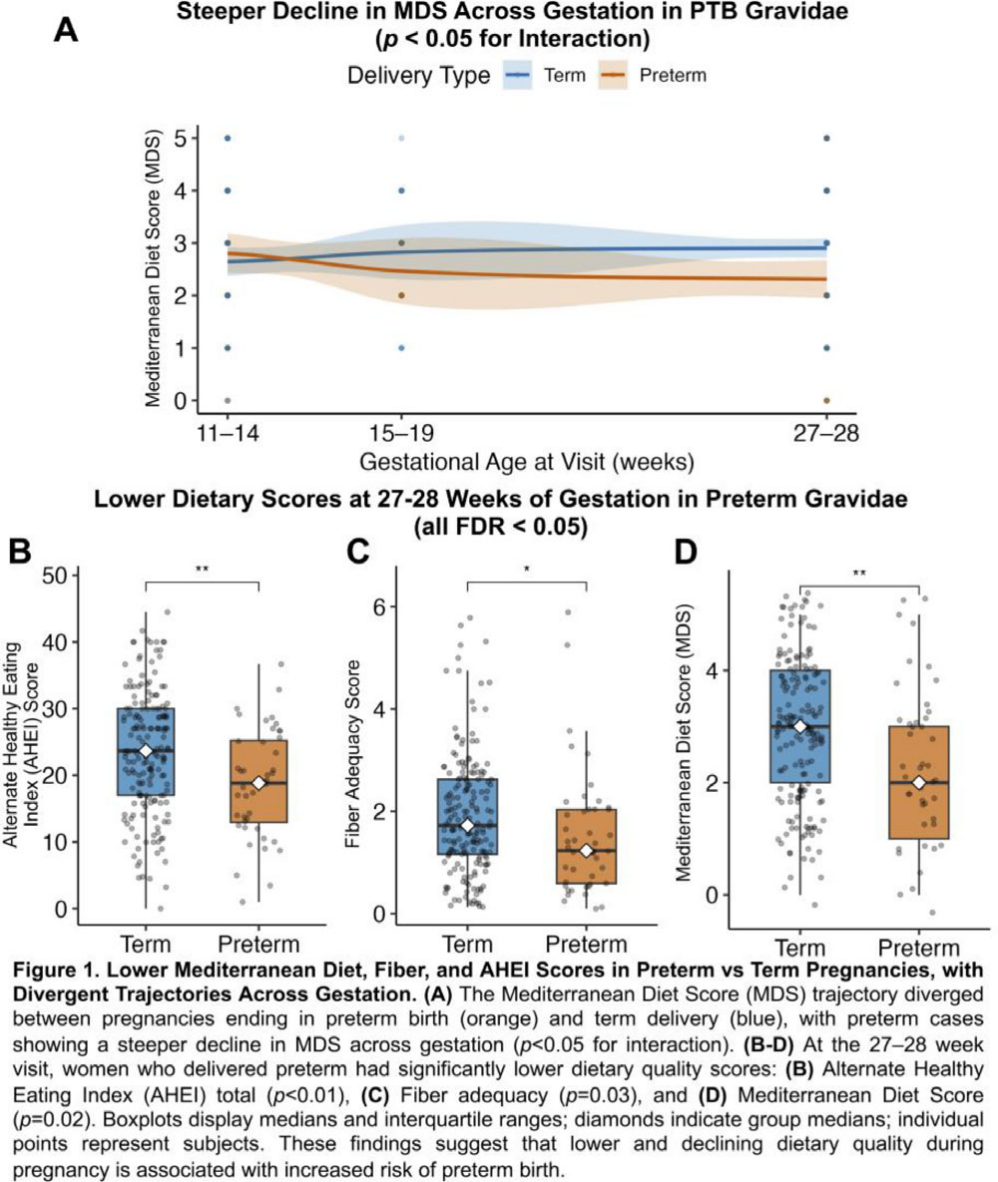

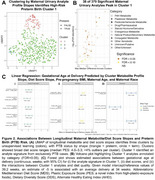

**2:45 PM – 3:00 PM**

## 52 | **Ultrasound Practice Benchmarks in Maternal Fetal Medicine Units in the United States**

Fadi Bsat^1^; Suwan Mehra^2^

^1^SMFM Practice Management Committee, West Springfield, MA; ^2^Advocate Children's Hospital, Advocate Childrens Hospital, IL

**Objective**: To establish national benchmarks for ultrasound practices in Maternal Fetal Medicine (MFM) units in the United States through a comprehensive member survey.

**Study Design**: A 41‐question survey via SurveyMonkey^®^ was emailed to all 2096 regular U.S.‐based members of the Society for Maternal Fetal Medicine (SMFM) in January 2025. The survey gathered data on demographics, practice size, scheduling, scope of services, quality and compliance processes, equipment, reporting software, and timing of consultations based on ultrasound findings.

**Results**: We received 232 unique responses. Most respondents were affiliated with academic institutions, while 17% were in private practice. Learners were present in 77% of units, and 67% of respondents reported performing remote interpretations. The average time allocated for a detailed fetal anatomy survey was approximately 60 minutes. In practices with mixed scheduling and without additional clinical responsibilities, the median number of patients scheduled per 8‐hour shift was 8–9 per sonographer and 30–39 per MFM physician. Screening fetal echocardiography was performed by 64% of respondents, although only 28% interpreted diagnostic studies for suspected cardiac anomalies. First‐trimester fetal anatomy surveys were performed by 42%, with an additional 39% planning implementation once billing challenges are resolved. Over 90% of significant ultrasound findings led to immediate consultations. The majority conducted quality (77%) and billing (74%) audits. Ultrasound equipment was predominantly purchased rather than leased, with equipment upgrades occurring within 5 years (45%) or every 5–10 years (44%). Ergonomic protocols aimed at reducing sonographer injury were widely reported.

**Conclusion**: These findings establish national benchmarks that can support MFM practices in evaluating and enhancing operational performance.

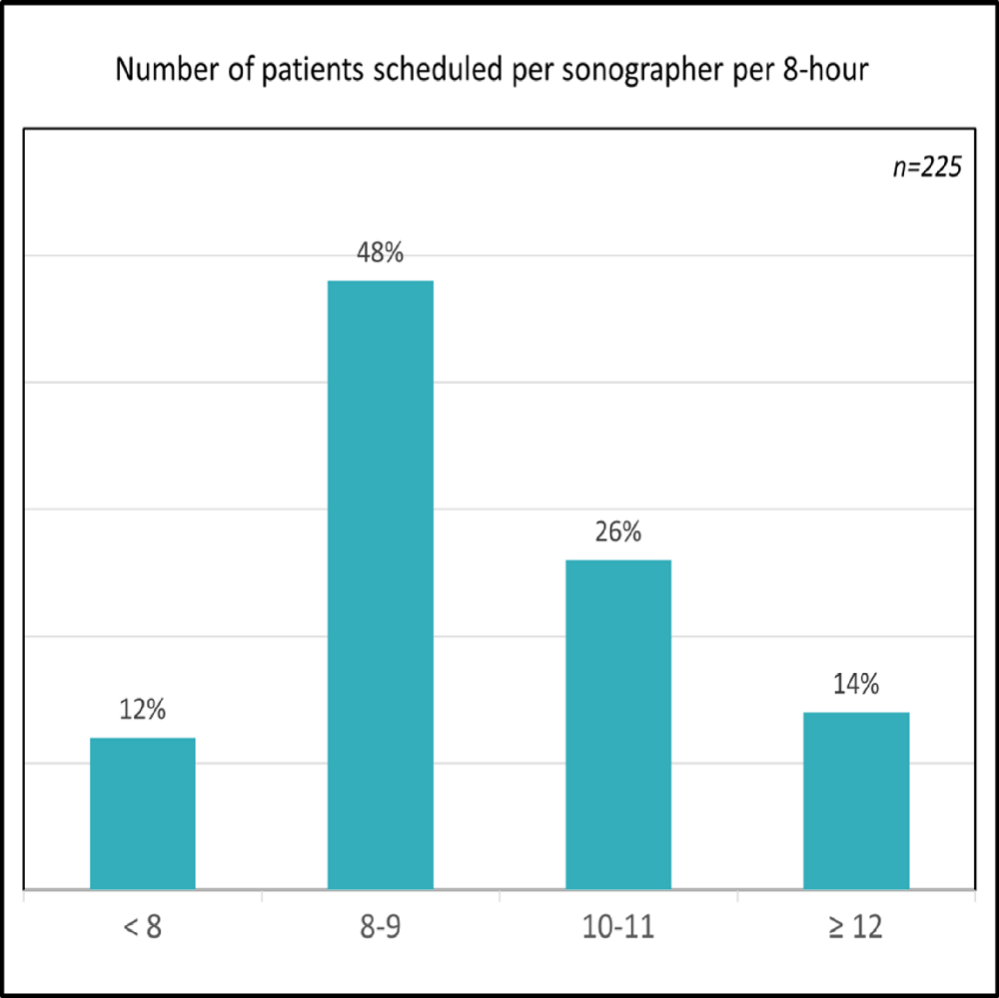

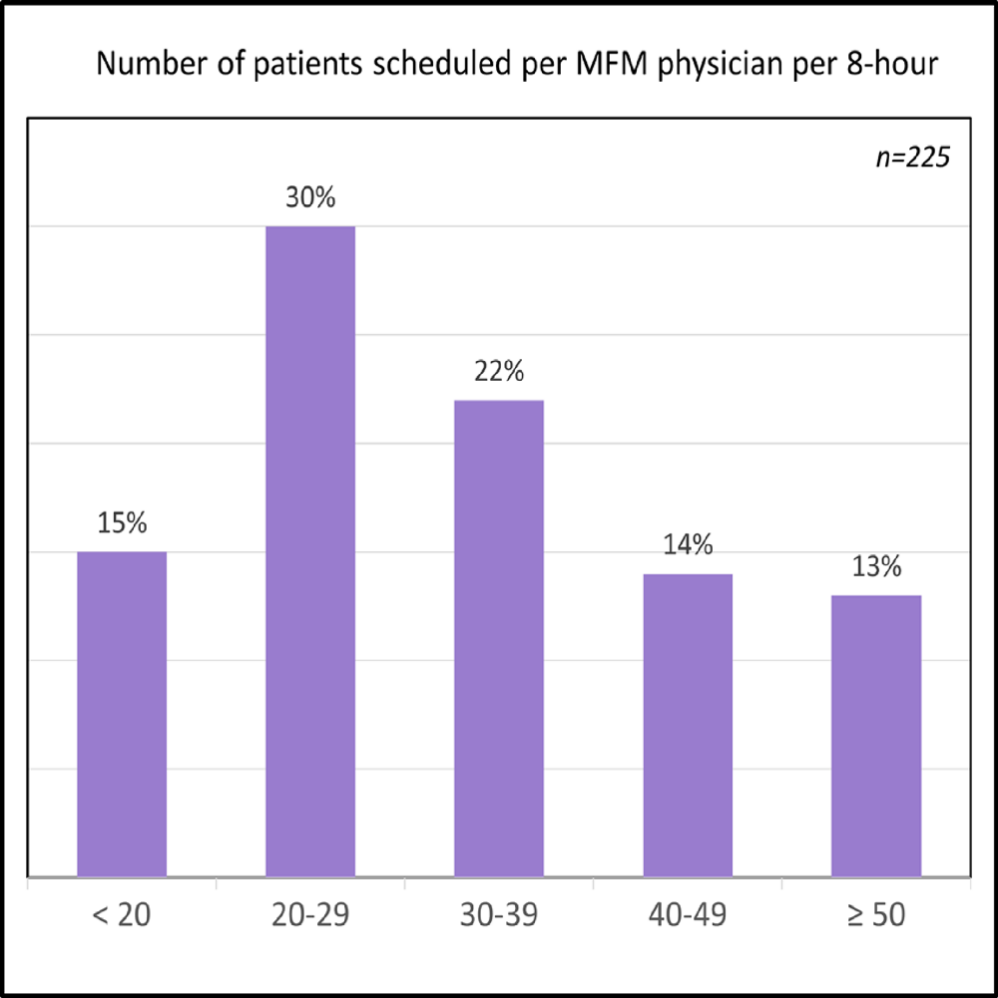

**3:00 PM – 3:15 PM**

## 53 | **Promotion of RSV Immunization Through Multiple Efforts (PRIME): A Sequential Multiple Assignment Randomized Trial**

Jeannie C. Kelly^1^; Abigail Arter^1^; Candice L. Woolfolk^1^; Elvin Geng^1^; Jason Newland^2^; Antonina I. Frolova^3^; Nandini Raghuraman^1^

^1^Washington University School of Medicine, Washington University School of Medicine, MO; ^2^Nationwide Children's Hospital, Nationwide Children's Hospital, OH; ^3^Washington University School of Medicine, St. Louis, MO

**Objective**: Persistent gaps in clinical care often require adaptive approaches that tailor intervention intensity based on individual response. Such strategies optimize efficiency by minimizing unnecessary intervention while escalating support for those who need more. To evaluate this approach for RSV vaccine uptake in pregnancy, we conducted the first individual‐level sequential multiple assignment randomized trial (SMART) in obstetrics.

**Study Design**: This was a Type 2 hybrid effectiveness‐implementation trial at a tertiary care center. Pregnant patients eligible for RSV vaccination at 28–30 weeks gestation were randomized to routine counseling (RC) or RC plus a visual aid (RC+VA). Patients unvaccinated by 34 weeks were re‐randomized to receive either a personalized electronic chart message from their physician or an educational video (EV) recommending vaccination (Figure). Co‐primary outcomes were RSV vaccine uptake (effectiveness) and implementation feasibility of recruitment, fidelity, and intervention delivery.

**Results**: Of 79 eligible patients, 50 (63%) were randomized (RC: *n* = 25; RC+VA; *n* = 25); baseline demographics were similar. All received their assigned first‐tier intervention. Eighteen (36%) were vaccinated by 34 weeks; no difference was seen in uptake between groups (RC: 44%, RC+VA: 28%; *p* = 0.239). Of the 32 who remained unvaccinated, 4 were lost to follow‐up and 28 were re‐randomized (personalized chart message: *n* = 14; video: *n* = 14), with fidelity of 100% for chart message and 79% for video. At 36 weeks, vaccine uptake was 36% for chart message and 43% for video. When analyzed by full adaptive sequence, 3 of 4 sequential strategies significantly outperformed first tier allocation alone, with vaccination rates of 72–79% vs. 28–44% (RRs 1.74–2.81) by 36 weeks (Table).

**Conclusion**: This SMART demonstrated both effectiveness and feasibility of adaptive strategies to improve maternal RSV vaccination uptake. Adaptive sequences almost doubled vaccine uptake compared to first tier alone. PRIME SMART supports the use of sequential, personalized interventions to address implementation gaps in obstetric care.